# Increased SPON1 promotes pancreatic ductal adenocarcinoma progression by enhancing IL‐6 trans‐signalling

**DOI:** 10.1111/cpr.13237

**Published:** 2022-04-29

**Authors:** Yanmiao Huo, Jian Yang, Jiahao Zheng, Dapeng Xu, Minwei Yang, Lingye Tao, Hongfei Yao, Xueliang Fu, Jianyu Yang, Dejun Liu, Rong Hua, Junfeng Zhang, Yongwei Sun, Lipeng Hu, Wei Liu

**Affiliations:** ^1^ Department of Biliary‐Pancreatic Surgery, Ren Ji Hospital, School of Medicine Shanghai Jiao Tong University Shanghai People's Republic of China; ^2^ State Key Laboratory of Oncogenes and Related Genes, Shanghai Cancer Institute, Ren Ji Hospital, School of Medicine Shanghai Jiao Tong University Shanghai People's Republic of China

## Abstract

**Objectives:**

This study investigated the specific molecular mechanism and the roles of extracellular matrix protein Spondin 1 (SPON1) in the development of pancreatic ductal adenocarcinoma (PDAC).

**Materials and Methods:**

The expression pattern and clinical relevance of SPON1 was determined in GEO, Ren Ji and TCGA datasets, further validated by immunohistochemical staining and Kaplan‐Meier analysis. Loss and gain of function experiments were employed to investigate the cellular function of SPON1 in vitro. Gene set enrichment analysis, luciferase assay, immunofluorescence and Western blot and immunoprecipitation were applied to reveal the underlying molecular mechanisms. Subcutaneous xenograft model was used to test the role of SPON1 in tumour growth and maintenance in vivo.

**Results:**

SPON1 is significantly upregulated in PDAC tumour tissues and correlated with progression of PDAC. Loss and gain of function experiments showed that SPON1 promotes the growth and colony formation ability of pancreatic cancer cells. Combining bioinformatics assays and experimental signalling evidences, we found that SPON1 can enhance the IL‐6/JAK/STAT3 signalling. Mechanistically, SPON1 exerts its oncogenic roles in pancreatic cancer by maintaining IL‐6R trans‐signalling through stabilizing the interaction of soluble IL‐6R (sIL‐6R) and glycoprotein‐130 (gp130) in PDAC cells. Furthermore, SPON1 depletion greatly reduced the tumour burden, exerted positive effect with gemcitabine, prolonging PDAC mice overall survival.

**Conclusions:**

Our data indicate that SPON1 expression is dramatically increased in PDAC and that SPON1 promotes tumorigenicity by activating the sIL‐6R/gp130/STAT3 axis. Collectively, our current work suggests SPON1 may be a potential therapy target for PDAC patient.

## Graphical abstract

Extracellular matrix protein spondin 1 is significantly upregulated in PDAC tumour cell, which exerts its oncogenic roles in pancreatic cancer by maintaining IL6R trans‐signalling through stabilizing the interaction of sIL6R and GP130 in PDAC cell, resulting in STAT3 signalling activating and tumour cell growth.

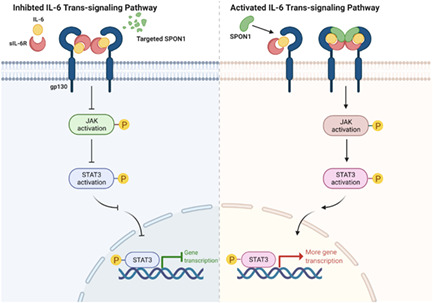

## 1 INTRODUCTION

Pancreatic cancer is one of the most deadly cancers, with a devastating median survival time of 6 months.^1^ In recent decades, despite progression in surgical treatment, chemotherapy and immune checkpoint therapy, the prognosis of pancreatic cancer patients is still not optimistic, and the 5‐year survival rate is only 8%.^2^, ^3^ Moreover, pancreatic cancer was the fourth leading cause of cancer‐related deaths in Europe in 2020^4^, ^5^ and is projected to become the second leading cause of cancer‐related deaths by 2030.^6^

The tumour microenvironment (TME), the complex environment in which tumour cells survive, is mainly composed of a variety of different extracellular matrix (ECM) components and stromal cells.^7^ Structural and non‐structural proteins are the main components of the ECM.^8^ Among them, non‐structural ECM proteins have many biological activities and play important roles in chronic inflammation, tumour growth, invasion and metastasis and immune microenvironment regulation.^9^, ^10^ Therefore, exploring the roles of ECM proteins in the occurrence and development of pancreatic cancer is expected to provide new insights into the diagnosis and treatment of pancreatic cancer.

The ECM protein SPON1, also known as F‐spondin or vascular smooth muscle cell growth‐promoting factor (VSGP), is a member of the thrombospondin family encoded by a highly conserved gene.^11^ SPON1 is a secreted ECM protein derived from the floor plate of vertebrate embryos. It was reported that SPON1 promotes the growth of neural axon cells while inhibiting the migration of neural crest cells.^12^, ^13^ In terms of disease research, SPON1 was regarded as a novel candidate hypertension gene, and its expression increased over time in spontaneously hypertensive rats.^14^ In addition, SPON1 could bind the amyloid precursor protein and inhibit its cleavage by β‐secretase, resulting in cognitive decline in Alzheimer's disease.^15^ SPON1 has been also reported to be abnormally expressed in solid tumours. Previous studies showed that SPON1 triggers focal adhesion kinase 1 (Fak) and tyrosine‐protein kinase Src signalling and promotes distant metastasis in osteosarcoma.^16^ Another study reported that SPON1 promotes tumour invasion and metastasis in liver cancer.^17^ However, these studies have not clearly elucidated the specific molecular mechanism, and the roles of SPON1 in the development of pancreatic cancer remain unknown.

In the present study, we discovered that SPON1 is markedly upregulated and correlated with malignant progression in PDAC. Functional experiments revealed that SPON1 silencing significantly inhibited the proliferation and colonization of PDAC cells. Mechanistically, SPON1 exerts its pro‐growth function in maintaining IL‐6R trans‐signalling by stabilizing the interaction of sIL‐6R/gp130 in PDAC cells. Using an in vivo PDAC model, we demonstrated that targeting SPON1 profoundly hindered the tumorigenesis of PDAC. More interestingly, the combination of gemcitabine treatment and SPON1 depletion more profoundly delayed tumour growth and significantly extended the survival of tumour‐bearing mice. Taken together, our results pave the way for developing novel therapeutic strategies for pancreatic cancer based on targeting SPON1.

## 2 MATERIAL AND METHODS

### 2.1 Cell culture and reagents

Human PDAC cell lines HPDE, AsPC‐1, BxPC‐3, Capan‐1, PANC‐1 and SW1990 were obtained from the Shanghai Cancer Institute, Ren Ji Hospital, School of Medicine, Shanghai Jiao Tong University. AsPC‐1 and BxPC‐3 were cultured in RPMI medium1640 supplemented with 10% foetal bovine serum (FBS) at 37°C in a humidified incubator under 5% CO_2_ condition, others were cultured in Dulbecco's modified Eagle's medium (DMEM) with 10% FBS. All cell lines underwent verification in January 2020 by Shanghai Cancer Institute and regular testing (every 3 months) to ensure no contamination with the Mycoplasma. Stattic (20 mM, S7024) was obtained from Selleck (Texas, USA), Puromycin (A1113802) was obtained from Gibco, recombinant human F‐Spondin/SPON1 Protein (3135‐SP) and IL‐6 (206‐IL) were obtained from R&D, Gemcitabine(LY‐188011)was purchased from Selleck.

### 2.2 Knockdown and overexpression assay

The lentivirus against *SPON1* was purchased from Gene Pharma (Shanghai, China), and the sequences targeting *SPON1* were: sh‐1, 5′‐GGGTGACTGACAAACCCATCT‐3′, sh‐2, 5′‐GGAGGAAGAAATTCGACAACA‐3′ (sense), and OE, NM_006108.4:183‐2606 Homo sapiens spondin 1 (SPON1) mRNA. LV16 (U6/Luciferase17&Puro) vector and LV17 (EF‐1a/Luciferase17&Puro) were used for SPON1 knockdown and overexpression experiments, respectively. For transducing lentivirus, the indicated cells were cultured in a six‐well plate, and 200 μl lentivirus suspension was added in the presence of 5 μg/ml polybrene (Gene Pharma, Shanghai, China). Forty‐eight hours after transduction, 5 μg/ml puromycin was added into culture medium for stable cell line screening.

### 2.3 Quantitative real‐time PCR

Total RNA was extracted using TRI REAGENT (MRC, TR118) according to the manufacturer instructions. The PrimeScript™ RT Master Mix (Takara, RR036A) was used to synthesize cDNAs in accordance with the protocol of the manufacturer. Quantitative real‐time PCR were performed with FastStart Universal SYBR Green Master (Roche, 04913914001) on a 7500 Real‐time PCR system (Applied Biosystems) at the recommended thermal cycling settings: one initial cycle at 95°C for 10 min followed by 40 cycles of 15 s at 95°C and 60 s at 60°C. Relative mRNA expression was calculated by the 2^−ΔΔCt^ method and normalized to *18S* mRNA levels. Primer sequences are listed as follows: *sIL‐6R* 5′‐CATGTGCGTCGCCAGTAGT‐3′, 5′‐AGCTCAAACCGTAGTCTGTAGA‐3′; *18S* 5′‐TGCGAGTACTCAACACCAACA‐3′, 5′‐GCATATCTTCGGCCCACA‐3′.

### 2.4 Western blotting

The lysates of protein samples were collected by using RIPA lysis and extraction buffer (ThermoFisher, 89900), separated by SDS‐PAGE in polyacrylamide gels, and transferred to nitrocellulose membranes. Subsequently, membranes were washed with TBST (50 mM TRIS + 150 mM sodium chloride + 0.1% Tween 20, pH 7.4) and blocked using 5% nonfat milk solution in TBST at least 1 h at room temperature. Membranes were then incubated with primary antibodies: ADAM10 (1:1000, Proteintech, 25900‐1‐AP), ADAM17 (1:1000, Abcam, b57484), IL‐6R (1:1000, Proteintech, 23457‐1‐AP), IL‐6R alpha (1:1000, R&D, MAB227), gp130 (1:1000, Proteintech, 67766‐1‐Ig), JAK2 (1:1000, Proteintech, 17670‐1‐AP), SPON1 (1:1000, Abcam, ab14271), STAT3 (1:1000, Proteintech, 60199‐1‐Ig), mIL‐6R (1:1000, MAB227, R&D), sIL‐6R (1:1000, HCA257, bio‐Rad), P‐JAK (1:1000, CST, 66245), P‐STAT3 (1:1000, CST, 9145S), β‐actin (1:1000, Abcam, ab8227). Molecular weight‐specific clipped bands were incubated with specific primary antibodies at 4°C overnight and were then incubated with HRP‐conjugated secondary antibodies (goat anti‐mouse, 1:10,000, Jackson ImmunoResearch, 115‐035‐003; goat anti‐rabbit, 1:10,000 Jackson ImmunoResearch, 111‐035‐003; goat anti‐chicken, 1:10,000, Abcam) for 1 h at room temperature. The bands were visualized with ECL reagents (ShareBio).

### 2.5 Cell Counting Kit‐8

For measurement of cell proliferation, indicated cells were seeded in plates (96‐well) at a moderate density. CCK‐8 reagent (10 μl/well, CCK‐8, Dojindo, Japan) mixed with the serum‐free medium (90 μl/well) was added to each well at 0, 1, 2, 3, and 4 days. After incubation for 1 h, the absorbance was measured at 450 nm using a Power Wave XS microplate reader (BIO‐TEK), and then the grow curve was plotted with the relative OD450 values on the vertical axis and the time on the horizontal axis. This experiment was repeated twice.

### 2.6 Colony‐formation assays

In the colony‐formation assays, indicated cells (3000 cells/ml) were seeded in six‐well plates. The colonies were collected after incubation for 2 weeks and then fixed with 4% paraformaldehyde fix solution and stained with 0.5% (w/v) crystal violet, followed by calculation with Image J. This experiment was repeated twice.

### 2.7 EdU (5‐ethynyl‐2′‐deoxyuridine)

The indicated cells were seeded into chambered coverslips (80826, ibidi) and incubated at 37°C with 5% CO_2_ condition for 48 h, and then added EdU working solution (10 μM, Yeasen) into culture medium at 37°C with 5% CO_2_ condition for overnight. After that, the chambers were fixed with 4% paraformaldehyde fix solution for 10 min at room temperature. After following the manufacturer's instruction, DAPI (G1012, Servicebio) were used to counterstain nuclei for 5 min. Confocal microscopes (Leica, Germany) were used to capture digital images.

### 2.8 Immunohistochemistry (IHC) assay

All the patients were supplied with written informed consent before enrollment, and the study was approved by the Research Ethics Committee of Ren Ji Hospital, School of Medicine, Shanghai Jiao Tong University. Scoring was calculated based on the percentage of positive‐staining cells: 0%–5% scored 0, 6%–35% scored 1, 36%–70% scored 2, and more than 70% scored 3; and staining intensity: no staining scored 0, weakly staining scored 1, moderately staining scored 2, and strongly staining scored 3. The final score was showed using the percentage score × staining intensity score as follows: “−” for a score of 0–1, “+” for a score of 2–3, “++” for a score of 4–6 and “+++” for a score of > 6. The median SPON1 expression was selected as the threshold to define low and high expression of SPON1. Low expression was defined as a total score < 4 and high expression with a total score ≧ 4. These scores were verified independently by two senior pathologists in a blinded manner. Specifically, positive staining in the endocrine pancreas was excluded for scoring. Specific antibodies used for immunohistochemistry were: SPON1 (1:100, Abcam, ab14271), PCNA (1:500, CST, 13110), P‐JAK (1:100, CST, 66245), P‐STAT3 (1:100, CST, 9145S).

### 2.9 Immunoprecipitation

Cells with indicated treatments were lysed in IP buffer supplemented with protLytic protease and phosphatase inhibitor cocktail, EDTA‐Free, 100× in DMSO (NCM, P002). Then lysates were incubated with pre‐linked anti‐antibody (5 μg, Abcam, ab40797), or control rabbit IgG (5 μg, Abcam, ab172730) Dynabeads protein G (Life technologies, 10004D) for 2 h at RT, following immunoblot with SPON1 (1:1000, Abcam, ab14271), gp130 (1:1000, Proteintech, 67766‐1‐Ig), sIL‐6R (1:1000, MAB227, R&D).

### 2.10 Immunofluorescence

The indicated cells were seeded into chambered coverslips (80826, ibidi) and incubated at 37°C with 5% CO_2_ condition for 48 h. After that, the chambers were fixed with 4% paraformaldehyde fix solution for 10 min at room temperature (necessarily, treated with 0.1% Triton X‐100 for 5 min at RT). After blocking with 5% BSA for 1 h, the chambered coverslips were incubated with primary antibodies: P‐STAT3 (1:100, CST, 9145S), IL‐6R (1:100, Proteintech, 23457‐1‐AP), gp130 (1:100, Proteintech, 67766‐1‐Ig), SPON1 (1:100, Abcam, ab14271) for overnight at 4°C, followed by incubation with secondary antibodies goat anti‐mouse FITC (1:200, Servicebio, GB22301), goat anti‐rabbit FITC (1:200, Servicebio, GB22303), goat anti‐mouse Cy3 (1:200, Servicebio, GB21301), goat anti‐mouse Cy3 (1:200, Servicebio, GB21303), or goat anti‐chicken 647 (1:200, Abcam, ab15075) for 1 h at RT. DAPI (G1012, Servicebio) were used to counterstain nuclei for 5 min. Confocal microscopes (Leica, Germany) were used to capture digital images.

### 2.11 Measurement of sIL‐6R

For measurement of sIL‐6R level in the culture medium, a total of 5 × 10^6^ indicated PDAC cells were seeded into 10 cm‐plates and allowed to attach overnight. Then cells were incubated for another 12 h in a humidifier at 37°C, and the culture medium was collected. For preparation of tissue homogenates, 14 PDAC tissues and paired non‐tumour tissues were washed with PBS to remove blood components. Following homogenization in a standard ELISA buffer, the tubes were centrifuged at 12,000 *g* for 30 min at 4°C. The supernatant was carefully removed and transferred for further measurement of sIL‐6R. For preparation of platelet‐poor‐plasma samples, 10 ml blood was collected into an EDTA plasma tube and centrifuged at 1000 *g* for 30 min at 4°C. Then plasma was aliquoted, labelled and stored at −80°C until measurement. All necessary precautions were taken to ensure complete certification of very low concentration of sIL‐6R in plasma. No patient was taking medication that could interfere with sIL‐6R metabolism. The amount of sIL‐6R in the culture medium, tissue homogenates and plasma was detected using a commercial ELISA kit according to the manufacturer's instructions (Elabscience, China). Values from each sample in tissues were normalized to total protein content as detected by BCA assay (Thermo Pierce).

### 2.12 Luciferase assay

As the characteristics of chemiluminescence reaction using the combination of luciferase and substrate, the indicated cells were transfected with vectors, STAT2, STAT3 and hedgehog using jetPRIME (Polyplus transfection). Next, the cells were assayed for firefly luciferase activities via a luciferase system (Promega, Madison, WI, USA) according to the manufacturer's instructions.

### 2.13 Animal model studies

Animal experiments were approved by Institutional Animal Care and Use Committee of East China Normal University (Shanghai, P.R. China). Mice were manipulated and housed according to the criteria outlined in the Guide for the Care and Use of Laboratory Animals prepared by the National Academy of Sciences and published by the NIH (Bethesda, MD).

### 2.14 Subcutaneous and orthotopic xenograft model

Athymic male nu/nu mice aged from 6 to 8 weeks were used in this study. Subcutaneous implant models were established by subcutaneous injection at a total cell number of 2 × 10^6^ for either shNC or shSPON1 PANC‐1 cells in 100 μl DMEM in the right back flank of mice. Tumour diameters were monitored with callipers every 3 days. Mice were sacrificed after 30 days and the tumour was isolated and weighed. Tumour volumes were calculated by volume = 0.5 × length × width.^2^ For orthotopic xenografts study, 1 × 10^6^ luciferase‐expressing Panc02 cells suspended in 25 μl DMEM were transplanted into the body of pancreas. Mice were randomly divided into four groups treated with 0.9% NaCl, shSPON1 cells, gemcitabine (50 mg/kg, every 5 days), and shSPON1 cells plus gemcitabine for 4 weeks after 5 days postsurgery, respectively. Luciferin emission imaging of isoflurane‐anesthetized animals was measured every 5 days using the IVIS spectrum (Calliper Life Sciences) after intraperitoneal injection of d‐luciferin (150 mg; Promega, catalogue no. P1043) into the mice. Five mice from each group were chosen randomly for bioluminescent imaging. Emission was quantified using Living Image software, version 4.5.3.

### 2.15 Data mining and bioinformatics analysis

Two Gene Expression Omnibus (GEO) datasets (GSE15471, GSE102238) were used in this study to determine the SPON1 expression in PDAC compared with the corresponding adjacent pancreatic tissues. The gene expression data for PDAC was downloaded from The Cancer Genome Atlas (TCGA). And our reproduction abided by the rules of the TCGA request. Gens set enrichment analysis (GSEA) was performed on the Broad Institute Platform and statistical significance (false discovery rate, FDR) was set at 0.25. The gene sets divided into the high‐SPON1 and low‐SPON1 groups were used to identify the differences. The gene expression was obtained using GEPIA2 database (http://gepia2.cancer-pku.cn/#analysis) based on TCGA cohort.

### 2.16 Statistical analysis

Numerical data processing was performed with GraphPad Prism 8.4.3 and Excel. Statistical analyses were done using SPSS 23.0 for Windows (IBM). Cumulative survival time was calculated by the Kaplan–Meier method and analysed by the log‐rank test. Correlation of SPON1 expression with categorical clinical variables in patients with PDAC was evaluated by *χ* ^2^ test or Fisher's exact test. Univariate and multivariate Cox regression analyses were performed to identify the factors that had a significant influence on survival by Cox proportional hazards model. The student's *t*‐test or one‐way ANOVA was used for comparison between groups. Values of *p* < 0.05 were considered statistically significant.

All error bars in this study represent the mean ± SD, except for bioluminescent emission, whose error bars represent the mean ± SEM (n.s, *p* > 0.05; *p < 0.05; **p < 0.01; ***p < 0.001).

## 3 RESULTS

### 3.1 SPON1 is upregulated in PDAC and associated with the poor prognosis of PDAC

**FIGURE 1:**
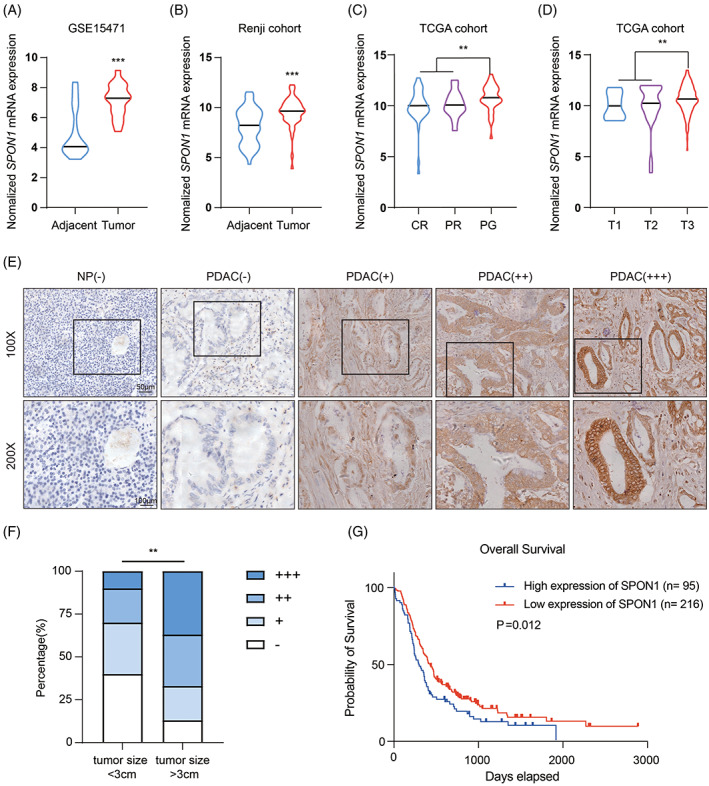
High SPON1 expression is correlated with poor prognosis in patients with PDAC. (A,B) *SPON1* expression in tumour tissues and paired corresponding adjacent pancreatic tissues in the GSE15471 and Ren Ji cohort datasets (GSE102238). (C,D) Upregulated expression of *SPON1* is linked to PDAC progression in TCGA database. (E) Standard immunohistochemical scoring of SPON1 expression in 311 pancreatic cancer tumours and corresponding adjacent normal tissues based on images. Scale bar, 50 μm. (F) The percentage of tumour size in SPON1 high and low expression group. (G) Kaplan–Meier analysis of overall survival related to the expression of SPON1 in 311 cases based on the Ren Ji cohort. **p < 0.01, ***p < 0.001

To determine the expression of *SPON1* in PDAC, we first analysed two related Gene Expression Omnibus (GEO) datasets: GSE15471 and the Ren Ji cohort (GSE102238). *SPON1* mRNA expression was upregulated in cancer tissues compared with the corresponding adjacent pancreatic tissues (Figure 1A,B). Next, we analysed the association of *SPON1* expression and clinical attributes in The Cancer Genome Atlas (TCGA) PDAC patient cohort. We found that *SPON1* expression was associated with the sensitivity of PDAC to chemotherapy. *SPON1* expression in the progressive (PG) group was significantly higher than that in the complete remission (CR) and partial remission (PR) groups (Figure 1C). In addition, we found that *SPON1* expression increased with increasing T stage, and *SPON1* expression increased sequentially in T1, T2 and T3 PDAC specimens (Figure 1D). To further validate our results at the protein level, we performed immunohistochemical (IHC) staining. Expression was scored based on staining intensity and area. As shown in Figure 1E, a higher staining score was exhibited by the tumour tissues. According to clinical data obtained from Ren Ji cohort, our results suggested that samples which are larger than 3 cm in size show higher staining score than the less 3 cm (Figure 1F). As expected, Kaplan–Meier analysis revealed that patients with higher SPON1 expression had a poorer prognosis (Figure 1G). These data suggested that SPON1 plays an important role as an oncogene in PDAC progression.

### 3.2 SPON1 promoted the growth of PDAC cell in vitro

**FIGURE 2:**
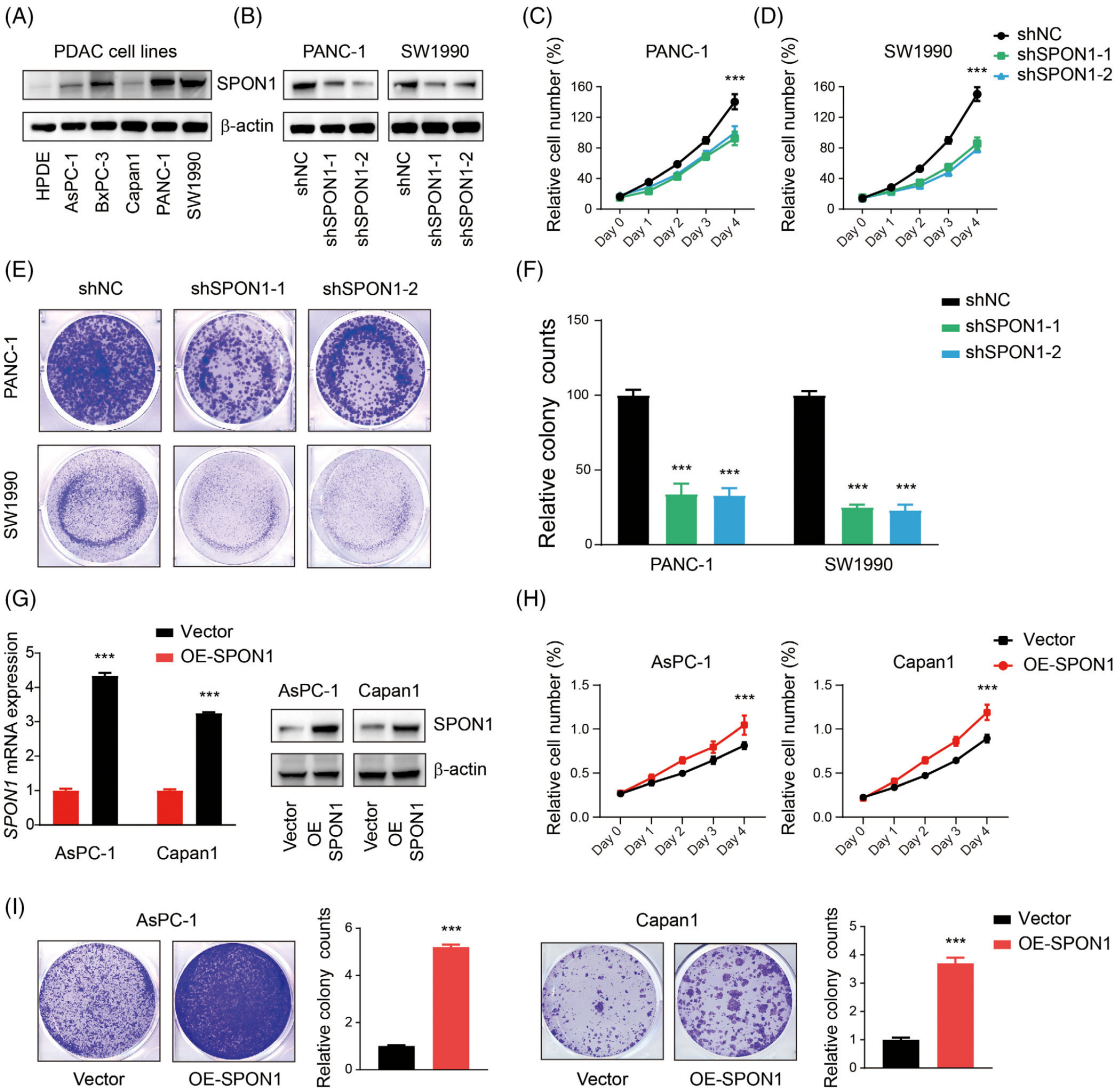
PDAC cell growth in vitro was affected by the level of SPON1 expression. (A) SPON1 expression in six pancreatic cancer cell lines at the protein level. (B) Protein expression of SPON1 in SPON1‐knockdown (SPON1^*KD*^) cells transfected with shRNA. (C,D) Cell Counting Kit‐8 assays showed that the proliferation of PANC‐1 and SW1990 cells was suppressed by SPON1 knockdown. (E) Colony‐formation assays showed that SPON1^*KD*^ cells exhibited suppressed proliferation. (F) Relative colony counts in SPON1^*KD*^ cells. (G) Relative mRNA and protein expression of SPON1 in SPON1‐overexpressing AsPC‐1 and Capan1 (SPON1^*OE*^) cells. (H,I) Relative cell proliferation of SPON1^*OE*^ cells expressing vector or overexpressing SPON1. ***p < 0.001. (ANOVA for C, D and H; two‐tailed Student *t* test for F, G and I)

To gain further insight into the roles of SPON1 in PDAC, we examined SPON1 expression in six pancreatic cancer cell lines at the protein level (Figure 2A). We selected PANC‐1 and SW1990 cells, which exhibit relatively high SPON1 expression, for genetic inhibition of SPON1 by short hairpin RNA (shRNA) transfection. After verifying SPON1 expression at the protein level (Figure 2B), cell proliferation was assessed. It was shown that knockdown of SPON1 expression impaired cell growth in vitro (Figure 2C–F). Meanwhile, we selected two cell lines that express SPON1 at relatively lower levels for SPON1 overexpression, and SPON1 expression was examined at the mRNA and protein levels (Figure 2G). As expected, cell proliferation and colony formation were enhanced by SPON1 overexpression (Figure 2H–I). The above results indicated that the level of SPON1 expression has crucial effects on PDAC cell growth.

### 3.3 SPON1 activates the IL‐6/JAK/STAT3 pathway in PDAC cells

**FIGURE 3:**
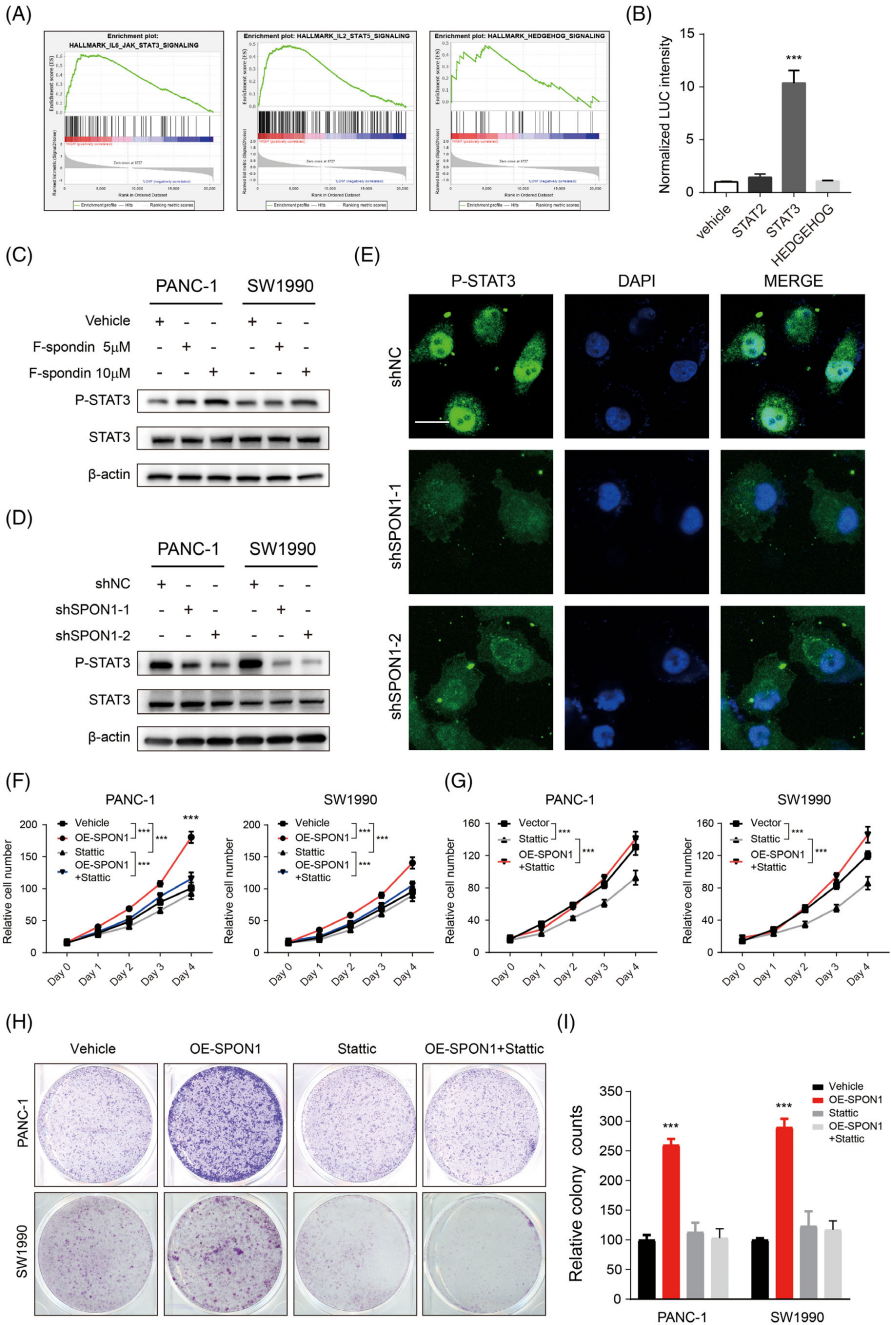
SPON1 activation enhances PDAC cell growth via IL‐6/JAK/STAT3. (A) Gene set enrichment analysis (GSEA) of the groups expressing SPON1 at high and low levels in three datasets was carried out by using hallmark gene sets. (B) Luciferase assay showed the normalized LUC intensities in vehicle, STAT2, STAT3 and HEDGEHOG cells. (C,D) PANC‐1 and SW1990 cells (C) and SPON1^*KD*^ cells (D) were cultured for 24 h with or without 5 μM or 10 μM F‐spondin. Phosphorylated and total STAT3 was measured by immunoblotting. (E) Immunofluorescence analysis of P‐STAT3 in SPON1^*KD*^ cells. Scale bar, 50 μm. (G) After treatment with plasmid for SPON1 overexpression with or without Stattic, the relative number of SPON1^*KD*^ cells was measured. (F,H,I) Cell proliferation of the indicated cells treated with vehicle, a plasmid for SPON1 overexpression, 5 μM Stattic (a selective STAT3 antagonist) or the plasmid for SPON1 expression plus 5 μM Stattic. ***p < 0.001 (two‐tailed Student *t* test for B and I; ANOVA for F and G)

To further understand the underlying mechanism by which SPON1 promotes PDAC cell proliferation, we first performed gene set enrichment analysis (GSEA) of samples in TCGA divided into two groups based on *SPON1* expression. As shown in Figure 3A, the HALLMARK_IL2_STAT5_SIGNALLING, HALLMARK_IL6_JAK_STAT3_SIGNALLING and HALLMARK_HEDGEHOG_SIGNALLING datasets were sifted. Further, luciferase assay was performed to figure out which pathway may mediate the pro‐growth effect of SPON1. The results showed that JAK‐STAT3 pathway activation was much higher than others pathway upon SPON1 overexpression in PDAC cell (Figure 3B). To verify the effects of SPON1 on this pathway, we then examined the protein level of phospho‐STAT3 in the nuclear (hereafter referred to as P‐STAT3) after treatment with different doses of F‐spondin. The results showed that the P‐STAT3 expression level increased with increasing doses of F‐spondin (Figure 3C–D). In general, phosphorylated STAT3 in the cytosol exerts its effects by entering the nuclei. Therefore, the nuclear translocation of P‐STAT3 was assessed by confocal microscopy and clearly decreased in the SPON1‐knockdown (SPON1^*KD*^) cell lines (Figure 3E). To validate whether SPON1 enhances cell proliferation via STAT3 activation, we used Stattic, a small molecule that specifically inhibits STAT3 activation and nuclear translocation, with IC50 dose 5 μM to suppress STAT3 activation in cell proliferation assays. The results indicated Stattic treatment diminished the growth promotive effect mediated by SPON1 overexpression (Figure 3F–I). Our data showed that SPON1 facilitates cell growth by activating STAT3.

### 3.4 SPON1 activated IL‐6 trans‐signalling to triggering IL‐6/JAK/STAT3 pathway

**FIGURE 4:**
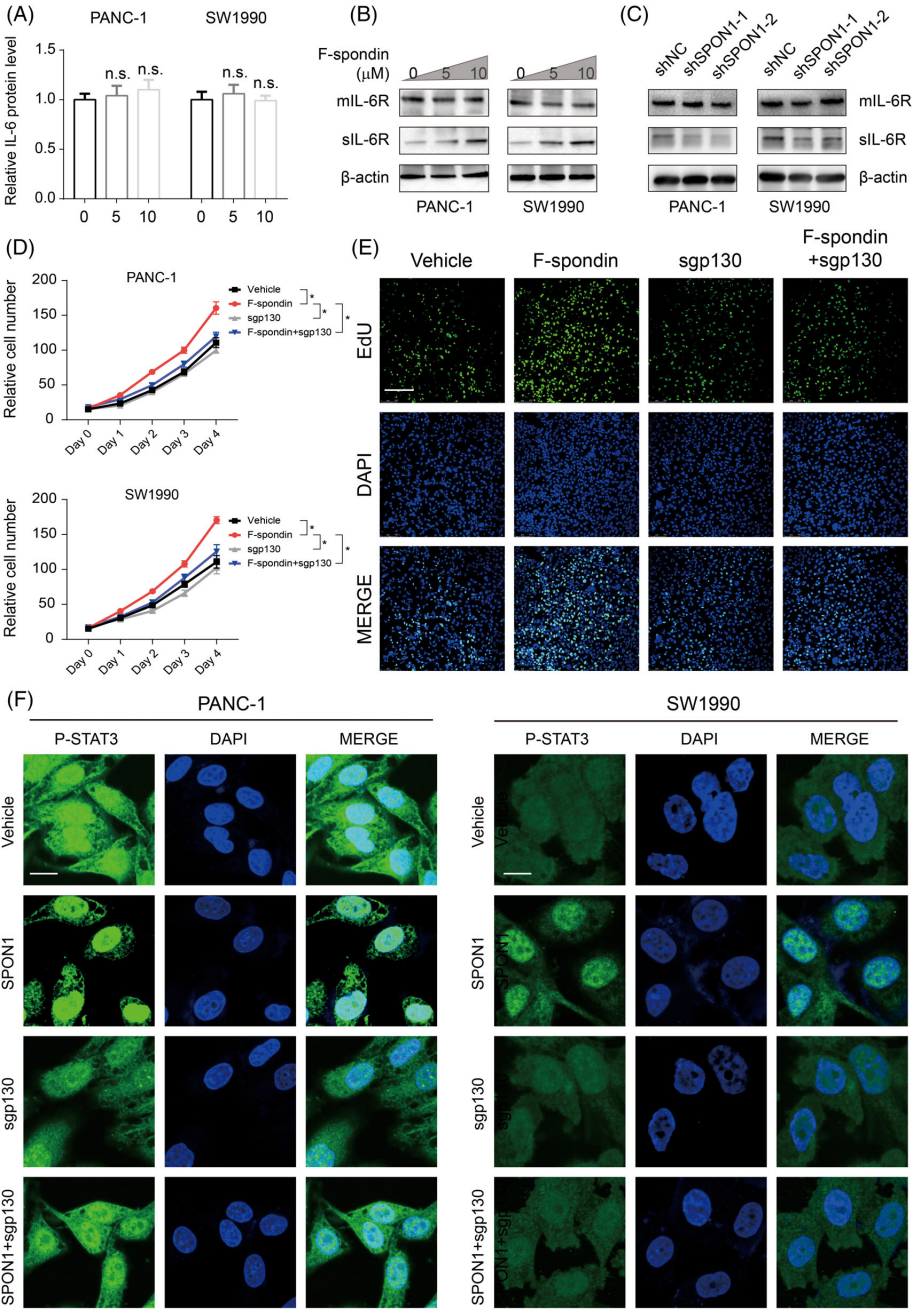
SPON1 plays important roles in IL‐6 trans‐signalling. (A) Relative IL‐6 protein levels in SPON1^*KD*^ cells were measured by ELISA (*n* = 3). (B) Immunoblot analysis of the indicated cells treated with 5 μM or 10 μM F‐spondin. (C) sIL‐6R and mIL‐6R protein levels in SPON1^*KD*^ cells were measured by western blotting. (D) Relative numbers of PDAC cells treated with vehicle, 10 μg/ml F‐spondin, 20 μg/ml sgp130 or F‐spondin plus sgp130. (E) EdU assays showed that SPON1 counteracted sgp130‐induced suppression of cell proliferation Scale bar, 50 μm. (F) Immunofluorescence staining for P‐STAT3 (green) in PANC‐1 and SW1990 cells upon indicated treatment. Scale bar, 50 μm. n.s. >0.05 (ANOVA for A; two‐tailed Student *t* test for D)

IL‐6 activates downstream signalling pathways by forming complexes with its receptors, including two subunits: IL‐6R (also called IL‐6α or CD126) and signal‐transducing glycoprotein‐130 (also called gp130, IL‐6β, CD130). In addition, IL‐6 signalling pathway models may vary in different contexts: in the classic IL‐6 signalling pathway, extracellular IL‐6 binds membrane‐bound IL‐6R (mIL‐6R) to yield a complex to which gp130 binds, forming a complex consisting of two IL‐6, two IL‐6R and two gp130 molecules^18^; alternativly, IL‐6 trans‐signalling that follows the classical pathway except that IL‐6 binds soluble IL‐6R (sIL‐6R) rather than mIL‐6R can also occur.^19^ The third model, termed IL‐6 trans‐presentation, has recently been identified and is specific to dendritic cells.^20^ IL‐6 level was measured by ELISA assays after administration of F‐spondin, and were not significantly different (Figure 4A). As shown in Figure 4B, we examined the IL‐6 related receptors after treatment with F‐spondin. It was shown that the level of sIL‐6R was markedly increased, while the mIL‐6R level was not. In addition, SPON1^*KD*^ cells showed reduced sIL‐6R protein levels; however, mIL‐6R levels were not reduced (Figure 4C).

To further explore the potential mechanism of SPON1‐induced STAT3 phosphorylation, we then performed cell proliferation assays to determine which model is responsible for this effect. Soluble gp130 (sgp130) blocks IL‐6 trans‐signalling by binding the IL‐6/sIL‐6R.^21^ Further, the PDAC cell lines were treated with F‐spondin, sgp130 or F‐spondin plus sgp130. The results revealed that sgp130 impaired cell proliferation. Also, sgp130 could diminish the F‐spondin‐induced proliferation advantage (Figure 4D,E). In line with this, the immunofluorescence results showed that F‐spondin could promote the nuclear translocation of p‐STAT3, which was impaired by sgp130 treatment (Figure 4F). We speculated that SPON1 activates the IL‐6 trans‐signalling pathway by competitive binding with sgp130. That is, sIL‐6R may be affected by SPON1 in some ways, resulting in changes to cell proliferation.

### 3.5 SPON1 enhances the IL‐6/JAK/STAT3 pathway by stabilizing the sIL‐6R/gp130 complex

**FIGURE 5:**
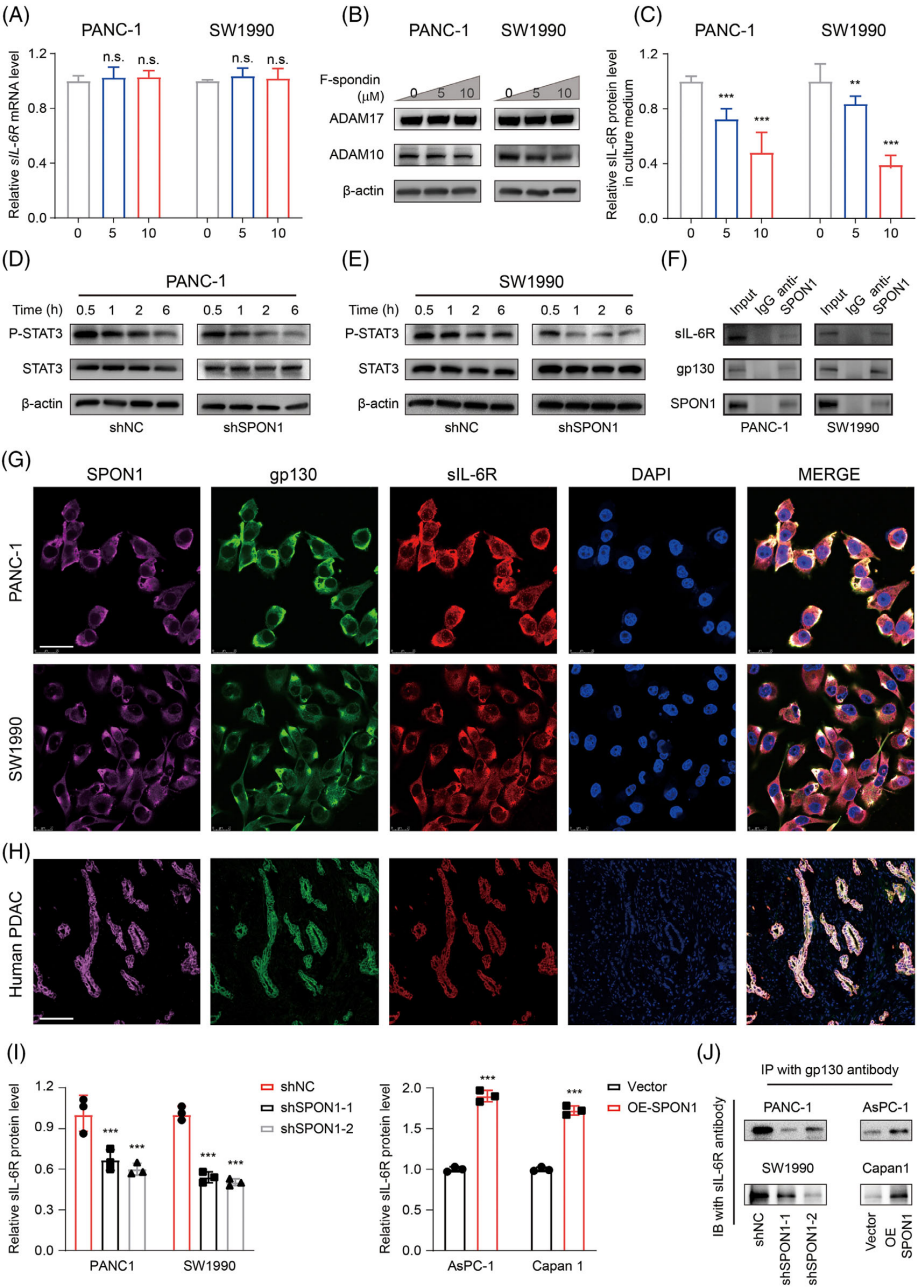
SPON1 cooperates with sIL‐6R/gp130 for signal transduction. (A) Relative *sIL‐6R* mRNA levels in PANC‐1 and SW1990 cells treated with 5 μM or 10 μM F‐spondin (*n* = 3). (B) Immunoblot analysis of ADAM10 and ADAM17 in PDAC cells treated with or without 5 μM or 10 μM F‐spondin. (C) sIL‐6R proteins derived from the indicated cell culture medium were measured by ELISA (*n* = 3). (D,E) SPON1^*KD*^ cells treated with IL‐6 for different times (30 min or 1, 2, or 6 h). Phosphorylated and total STAT3 was measured by immunoblotting. (F) Co‐immunoprecipitation of SPON1 and sIL‐6R/gp130 in PANC‐1 and SW1190 cells. (G) Immunofluorescence staining for SPON1 (pink), gp130 (green) and sIL‐6R (red) in PDAC cells. Scale bar, 50 μm. (H) Representative IHC images of SPON1, gp130 and sIL‐6R in the Ren Ji cohort PDAC specimen. Scale bar, 50 μm. (I) ELISA detection results of sIL‐6R protein level in the cell membrane subcellular fractionation in SPON1^*KD*^ and SPON1^*OE*^ cells (*n* = 3). (J) WB of sIL‐6R protein pull down by anti‐GP130 antibody in SPON1^*KD*^ and SPON1^*OE*^ cells. n.s. >0.05, **p < 0.01, ***p < 0.001 (two‐tailed Student *t* test for A, C and I)

We next investigated why sIL‐6R was increased with increasing F‐spondin doses. In general, sIL‐6R proteins were translated from alternatively spliced IL‐6R mRNA. Moreover, a disintegrin and metalloproteinase domain‐containing protein 10 (ADAM10) and metalloproteinase domain‐containing protein 17 (ADAM17) can cleave mIL‐6R to generate sIL‐6R.^22^ Therefore, we examined the mRNA level of *sIL‐6R* after the addition of F‐spondin, but the difference was not statistically significant (Figure 5A). Then, ADAM10 and ADAM17 were assessed at the protein level and showed no significant changes as well (Figure 5B). Intriguingly, F‐spondin decreased the sIL‐6R level in the culture medium (Figure 5C). The results of immunoblot analysis showed decreased P‐STAT3 levels after treatment with IL‐6 for increasing durations (Figure 5D,E). Given the above results, we speculated that SPON1 contributes to the formation of the sIL‐6R/gp130 complex, thus sustaining IL‐6 trans‐signalling and STAT3 activation. To test our hypothesis, we performed endogenous immunoprecipitation assays to confirm the interaction between SPON1 and sIL‐6R/gp130 (Figure 5F). Consistently, immunofluorescence co‐localization analysis and immunofluorescence staining were used to further verify the interaction between SPON1 and sIL‐6R/gp130 (Figures 5G and S1). The results showed that both SPON1 and sIL‐6R/gp130 were co‐localized in human PDAC tumour tissues (Figure 5H). Besides, we detected the changes of sIL‐6R located in cell membrane. The cell membrane of SPON1^*KD*^ cell and control cells were isolated by subcellular protein fractionation kit, following ELISA detection. As expected, the membrane location of sIL‐6R dramatically decreased upon SPON1 knockdown. The CO‐IP results showed that much less of sIL‐6R interacted with gp130 in SPON1^*KD*^ cells when compared with the control (Figure 5I–J). Conversely, extopic SPON1 expression could enhance the membrane location of sIL‐6R/gp130 and its interaction (Figure 5I,J). Taken together, these data showed that SPON1 stabilizes the sIL‐6R/gp130 complex to sustaining the IL‐6 trans‐signalling pathway.

### 3.6 Genetic inhibition of SPON1 suppressed the progression of PDAC in vivo

**FIGURE 6:**
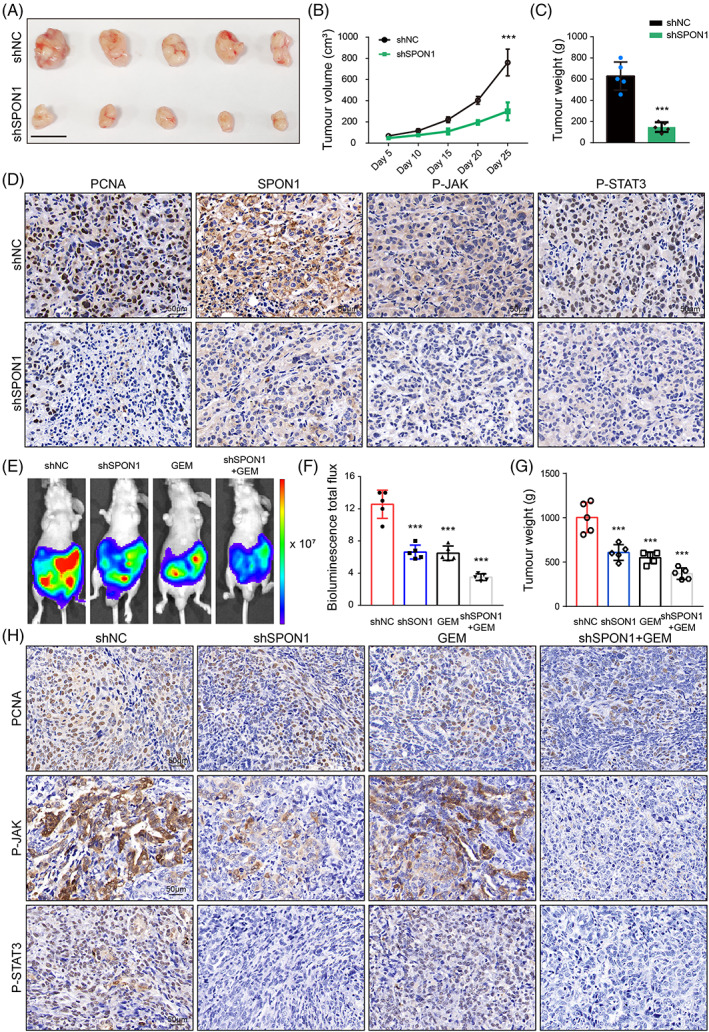
SPON1 promoted PDAC progression in vivo. (A–C) Growth of subcutaneous xenografts from SPON1^*KD*^ cells. Scale bar, 1 cm. (D) IHC staining of subcutaneous xenografts for PCNA, P‐JAK and P‐STAT3. Scale bar, 50 μm. (E) Representative bioluminescence image of mice orthotopically implanted with luciferase‐expressing PANC1 cells administered shNC, shSPON1, gemcitabine or shSPON1 in combination with gemcitabine every 5 days. (F) Bioluminescence total flux showed that the group treated with shSPON1 cells plus gemcitabine exhibited lower bioluminescence emission. (G) Tumour weight decreased with gemcitabine treatment. (H) IHC staining of mouse orthotopically implanted tissue for PCNA, P‐JAK and P‐STAT3. Scale bar, 50 μm. ***p < 0.001 (ANOVA for B; two‐tailed Student *t* test for C, F and G)

A subcutaneous xenograft model was used to further test the role of SPON1 in tumour growth and maintenance in vivo. As shown in Figure 6A–C, mice injected with SPON1^*KD*^ cells exhibited a lower tumour burden than the control group. Specifically, the results showed that the negative control (NC) mice developed larger tumours than the shSPON1 mice in terms of size, volume and weight. In addition, immunoreactivity for proliferation index proliferating cell nuclear antigen (PCNA), P‐JAK and P‐STAT3 was significantly reduced in SPON1^*KD*^ cells compared with the corresponding controls (Figures 6D and S2A).

Gemcitabine (GEM) can profoundly improve the prognosis of advanced pancreatic cancer, but the development of chemoresistance still leads to poor clinical outcomes. On the basis of treatment with GEM, more pancreatic cancer patients require precision treatment and targeted drugs as well. Therefore, we wondered whether SPON1 knockdown would have antitumour effects with GEM and then generated orthotopic PDAC mice model. Total luminescence flux derived from bioluminescence imaging was used to evaluate orthotopic tumour growth. The results showed that the group injected with SPON1^*KD*^ cells combined with gemcitabine exhibited lower bioluminescent emission and a greatly reduced tumour burden compared with injection with only SPON1^*KD*^ cells (Figure 6E–G). As expected, combination treatment further reduced the expression of PCNA, P‐JAK and P‐STAT3 (Figures 6H and S2B). Our data demonstrated that targeting SPON1 with gemcitabine effectively prevented PDAC progression.

## 4 DISCUSSION

Pancreatic ductal adenocarcinoma (PDAC), one of the most devastating malignancies, is characterized by the excessive deposition of extracellular matrix (ECM), termed desmoplasia.^23^ Altered expression of ECM components in the tumour microenvironment (TME) greatly regulates PDAC cancer cell growth and survival, adhesion and migration,^24^ indicating that targeting the ECM would greatly facilitate the design and development of novel therapeutic approaches for PDAC.

Previous studies have shown that SPON1 expression is dramatically increased in osteosarcoma and hepatocellular carcinoma (HCC).^16^, ^17^ Consistently, we found that SPON1 expression was significantly increased in PDAC specimens upon the analysis of pairs of normal and tumour tissues from GEO dataset GSE15471, and this finding was also confirmed by microarrays and tumour tissue arrays with the Ren Ji PDAC cohort at both the mRNA and protein levels. Taking use of genome‐scale screening, SPON1 has been reported to act as a biomarker of prostate cancer,^25^ ovarian cancer,^26^ gastric cancer^27^ and colorectal cancer.^28^ Additionally, we found that SPON1 expression was associated with tumour TNM stage and tumour size in PDAC. Interestingly, we found that PDAC patients with low SPON1 expression were more sensitive to chemotherapy. Moreover, we provide in vivo evidence that targeting SPON1 exerted a promotive effect with gemcitabine in orthotopic xenograft PDAC mice, providing insight into the treatment of PDAC patients with chemotherapy.

Although several studies have reported the dysregulation of SPON1 and its role in promoting tumour growth, the specific mechanism by which SPON1 affects cancer development remains largely unknown. F‐Spondin, an extracellular protein, mainly exerts its function by regulating intracellular signalling pathways. Heping et al. reported that SPON1 promotes osteosarcoma cell migration and invasion by triggering Fak and Src signalling.^16^ However, this study did not provide evidence regarding how Fak and Src signalling is activated. In addition, SPON1 was shown to promote survival in a murine neuroblastoma model under stressful conditions via a p38 MAPK dependent pathway, ultimately upregulating IL‐6 expression.^29^ Consistent with the above mentioned study, the details regarding SPON1‐mediated activation of p38 MAPK signalling have not been researched. In our current study, by combining bioinformatics assays and experimental evidence, we found that SPON1 can activate IL‐6/JAK/STAT3 signalling. However, our results show that SPON1 does not affect IL‐6 expression at either the mRNA or protein level. The effects of receptor blockade showed that the oncogenic effects of SPON1 depend on IL‐6 trans‐signalling, in which IL‐6R shedding plays important roles. Our research showed that IL‐6R shedding was not altered upon SPON1 knockdown and treatment. Further experiments showed that F‐spondin could interact with both sIL‐6R and gp130 and sustain IL‐6 trans‐signalling, resulting in continuous IL‐6/JAK/STAT3 signalling activation in PDAC cells. Our data indicated that SPON1 could interacted with sIL‐6R and gp130. However, the specific way that SPON1 exert its function needs more efforts. On the one side, SPON1 could combined with sIL‐6R and gp130. On the other side, SPON1, as a secreted ECM protein, also could interacted with sIL‐6R firstly in the condition media in vitro or extracellular compartment of PDAC tumour, then binding to the gp130. In addition, previous studies reported that IL‐6‐STAT3 signalling could crosstalk with IL2‐STAT5 pathway.^30^, ^31^ In line with this, our data showed that IL2‐STAT5 axis had some extent activation, not as stronger as IL‐6‐STAT3 signalling. In summary, our data reveal the roles of SPON1 in signalling and as a mediator of pancreatic cancer.

## 5 CONCLUSION

We have shown that SPON1 expression is dramatically increased in PDAC and that SPON1 promotes tumorigenicity by activating the sIL‐6R/gp130/STAT3 axis. We have also demonstrated that targeting SPON1 may represent an attractive therapeutic strategy. Collectively, our findings provide not only new insights into the role of SPON1 in cancer progression but also a novel and effective therapeutic approach for the eradication of PDAC, which remains a life‐threatening disease without an effective, targeted therapeutic strategy.

## CONFLICT OF INTEREST

The authors declare no competing interests.

## AUTHOR CONTRIBUTIONS

Wei Liu, Lipeng Hu, Yongwei Sun and Junfeng Zhang designed and supervised this study. Yanmiao Huo, Jian Yang, Jiahao Zheng, Minwei Yang, Lingye Tao, Hongfei Yao and Xueliang Fu conducted the experiments. Jian Yang drafted the manuscript. Yanmiao Huo, Dapeng Xu, Jianyu Yang, Dejun Liu and Rong Hua analysed data and performed the statistics. All authors approved the final version of the manuscript.

## Supporting information


**FIGURE S1** SPON1 do not co‐localize with mIL6R.
**FIGURE S2** Targeting SPON1 inhibits JAK‐STAT3 pathway in vivo (A,B). The statistical results of tumour section IHC staining intensity

## Data Availability

All above data that support the findings of this study are available from the authors upon reasonable request.

## References

[1] Mizrahi JD , Surana R , Valle JW , Shroff RT . Pancreatic cancer. Lancet. 2020;395(10242):2008‐2020. 32593337 10.1016/S0140-6736(20)30974-0

[2] Moore A , Donahue T . Pancreatic cancer. JAMA. 2019;322(14):1426. 31593274 10.1001/jama.2019.14699 PMC7021307

[3] Neoptolemos JP , Kleeff J , Michl P , Costello E , Greenhalf W , Palmer DH . Therapeutic developments in pancreatic cancer: current and future perspectives. Nat Rev Gastroenterol Hepatol. 2018;15(6):333‐348. 29717230 10.1038/s41575-018-0005-x

[4] Arnold M , Abnet CC , Neale RE , et al. Global burden of 5 major types of gastrointestinal cancer. Gastroenterology. 2020;159(1):335‐349.e315. 32247694 10.1053/j.gastro.2020.02.068 PMC8630546

[5] Quante AS , Ming C , Rottmann M , et al. Projections of cancer incidence and cancer‐related deaths in Germany by 2020 and 2030. Cancer Med. 2016;5(9):2649‐2656. 27356493 10.1002/cam4.767 PMC5055190

[6] Rahib L , Smith BD , Aizenberg R , Rosenzweig AB , Fleshman JM , Matrisian LM . Projecting cancer incidence and deaths to 2030: the unexpected burden of thyroid, liver, and pancreas cancers in the United States. Cancer Res. 2014;74(11):2913‐2921. 24840647 10.1158/0008-5472.CAN-14-0155

[7] Hinshaw DC , Shevde LA . The tumor microenvironment innately modulates cancer progression. Cancer Res. 2019;79(18):4557‐4566. 31350295 10.1158/0008-5472.CAN-18-3962 PMC6744958

[8] Karamanos NK , Piperigkou Z , Passi A , Gotte M , Rousselle P , Vlodavsky I . Extracellular matrix‐based cancer targeting. Trends Mol Med. 2021;27:1000‐1013. 34389240 10.1016/j.molmed.2021.07.009

[9] Chiodoni C , Colombo MP , Sangaletti S . Matricellular proteins: from homeostasis to inflammation, cancer, and metastasis. Cancer Metastasis Rev. 2010;29(2):295‐307. 20386958 10.1007/s10555-010-9221-8

[10] Li Z , Zhang Y , Liu Z , et al. ECM1 controls T(H)2 cell egress from lymph nodes through re‐expression of S1P(1). Nat Immunol. 2011;12(2):178‐185. 21217760 10.1038/ni.1983

[11] Zisman S , Marom K , Avraham O , et al. Proteolysis and membrane capture of F‐spondin generates combinatorial guidance cues from a single molecule. J Cell Biol. 2007;178(7):1237‐1249. 17875744 10.1083/jcb.200702184 PMC2064656

[12] Adams JC , Tucker RP . The thrombospondin type 1 repeat (TSR) superfamily: diverse proteins with related roles in neuronal development. Dev Dyn. 2000;218(2):280‐299. 10842357 10.1002/(SICI)1097-0177(200006)218:2<280::AID-DVDY4>3.0.CO;2-0

[13] Burstyn‐Cohen T , Frumkin A , Xu YT , Scherer SS , Klar A . Accumulation of F‐spondin in injured peripheral nerve promotes the outgrowth of sensory axons. J Neurosci. 1998;18(21):8875‐8885. 9786993 10.1523/JNEUROSCI.18-21-08875.1998 PMC6793537

[14] Clemitson JR , Dixon RJ , Haines S , et al. Genetic dissection of a blood pressure quantitative trait locus on rat chromosome 1 and gene expression analysis identifies SPON1 as a novel candidate hypertension gene. Circ Res. 2007;100(7):992‐999. 17332427 10.1161/01.RES.0000261961.41889.9c PMC3533402

[15] Sherva R , Tripodis Y , Bennett DA , et al.; GENAROAD Consortium, Alzheimer's Disease Neuroimaging Initiative, Alzheimer's Disease Genetics Consortium . Genome‐wide association study of the rate of cognitive decline in Alzheimer's disease. Alzheimers Dement 2014;10(1):45–52. 23535033 10.1016/j.jalz.2013.01.008 PMC3760995

[16] Chang H , Dong T , Ma X , et al. Spondin 1 promotes metastatic progression through Fak and Src dependent pathway in human osteosarcoma. Biochem Biophys Res Commun. 2015;464(1):45‐50. 26032498 10.1016/j.bbrc.2015.05.092

[17] Dai W , Huang HL , Hu M , et al. microRNA‐506 regulates proliferation, migration and invasion in hepatocellular carcinoma by targeting F‐spondin 1 (SPON1). Am J Cancer Res. 2015;5(9):2697‐2707. 26609477 PMC4633899

[18] Xiang DM , Sun W , Ning BF , et al. The HLF/IL‐6/STAT3 feedforward circuit drives hepatic stellate cell activation to promote liver fibrosis. Gut. 2018;67(9):1704‐1715. 28754776 10.1136/gutjnl-2016-313392

[19] Heinrich PC , Behrmann I , Haan S , Hermanns HM , Muller‐Newen G , Schaper F . Principles of interleukin (IL)‐6‐type cytokine signalling and its regulation. Biochem J. 2003;374(Pt 1):1‐20. 12773095 10.1042/BJ20030407 PMC1223585

[20] Heink S , Yogev N , Garbers C , et al. Trans‐presentation of IL‐6 by dendritic cells is required for the priming of pathogenic TH17 cells. Nat Immunol. 2017;18(1):74‐85. 27893700 10.1038/ni.3632 PMC5164931

[21] Lamertz L , Rummel F , Polz R , et al. Soluble gp130 prevents interleukin‐6 and interleukin‐11 cluster signaling but not intracellular autocrine responses. Sci Signal. 2018;11(550):eaar7388. 30279168 10.1126/scisignal.aar7388

[22] Riethmueller S , Somasundaram P , Ehlers JC , et al. Proteolytic origin of the soluble human IL‐6R in vivo and a decisive role of N‐glycosylation. PLoS Biol. 2017;15(1):e2000080. 28060820 10.1371/journal.pbio.2000080 PMC5218472

[23] Espinet E , Gu Z , Imbusch CD , et al. Aggressive PDACs show hypomethylation of repetitive elements and the execution of an intrinsic IFN program linked to a ductal cell of origin. Cancer Discov. 2021;11(3):638‐659. 33060108 10.1158/2159-8290.CD-20-1202 PMC9216338

[24] Hosein AN , Brekken RA , Maitra A . Pancreatic cancer stroma: an update on therapeutic targeting strategies. Nat Rev Gastroenterol Hepatol. 2020;17(8):487‐505. 32393771 10.1038/s41575-020-0300-1 PMC8284850

[25] Wang LY , Cui JJ , Zhu T , et al. Biomarkers identified for prostate cancer patients through genome‐scale screening. Oncotarget. 2017;8(54):92055‐92063. 29190897 10.18632/oncotarget.20739 PMC5696163

[26] Pyle‐Chenault RA , Stolk JA , Molesh DA , et al. VSGP/F‐spondin: a new ovarian cancer marker. Tumour Biol. 2005;26(5):245‐257. 16103746 10.1159/000087379

[27] Ding Y , Chen Y , Wu M , et al. Identification of genes associated with gastric cancer survival and construction of a nomogram to improve risk stratification for patients with gastric cancer. Oncol Lett. 2020;20(1):215‐225. 10.3892/ol.2020.11543 PMC7291675 32537023

[28] Tamjidifar R , Akbari M , Tarzi S , et al. Prognostic and diagnostic values of miR‐506 and SPON 1 in colorectal cancer with clinicopathological considerations. J Gastrointest Cancer. 2021;52(1):125‐129. 31927744 10.1007/s12029-019-00356-0

[29] Cheng YC , Liang CM , Chen YP , Tsai IH , Kuo CC , Liang SM . F‐spondin plays a critical role in murine neuroblastoma survival by maintaining IL‐6 expression. J Neurochem. 2009;110(3):947‐955. 19549008 10.1111/j.1471-4159.2009.06186.x

[30] Tormo AJ , Letellier MC , Sharma M , Elson G , Crabe S , Gauchat JF . IL‐6 activates STAT5 in T cells. Cytokine. 2012;60(2):575‐582. 22854263 10.1016/j.cyto.2012.07.002

[31] Rani A , Murphy JJ . STAT5 in cancer and immunity. J Interferon Cytokine Res. 2016;36(4):226‐237. 26716518 10.1089/jir.2015.0054

