# Supplementary material for: Increased SPON1 promotes pancreatic ductal adenocarcinoma progression by enhancing IL‐6 trans‐signalling
Source: Cell Prolif. 2022 Apr 29;55(5):e13237. doi: 10.1111/cpr.13237 (PMC9136514; doi:10.1111/cpr.13237)
Supplement: Supplementary file 1 — FIGURE S1 SPON1 do not co‐localize with mIL6R. FIGURE S2 Targeting SPON1 inhibits JAK‐STAT3 pathway in vivo (A,B). The statistical results of tumour section IHC staining intensity [file CPR-55-e13237-s001.docx]

Supplementary figure 1


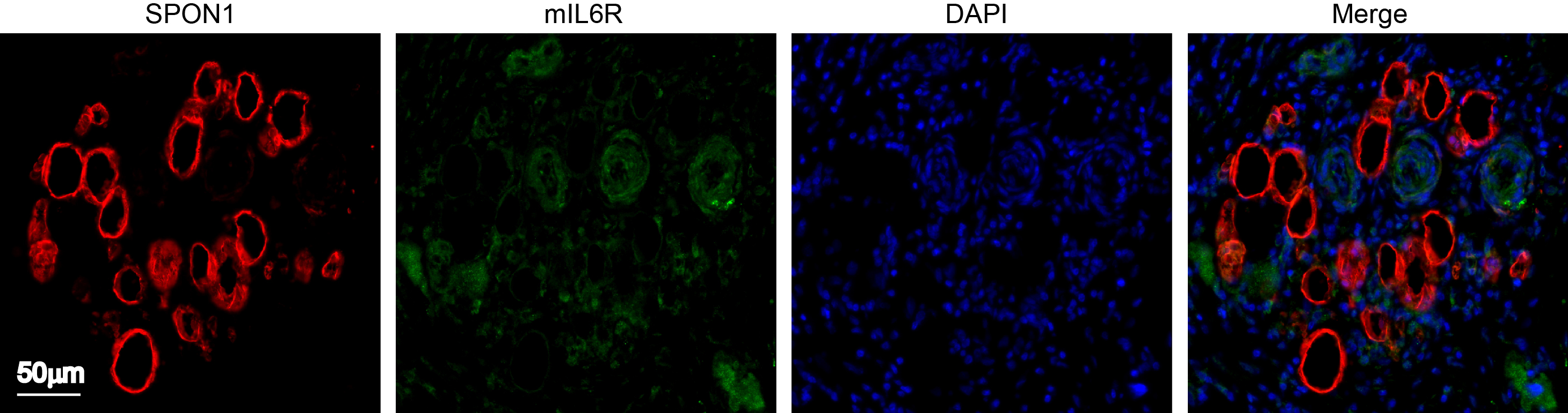


Supplementary figure 1 SPON1 do not co-localize with mIL6R.

Supplementary figure 2


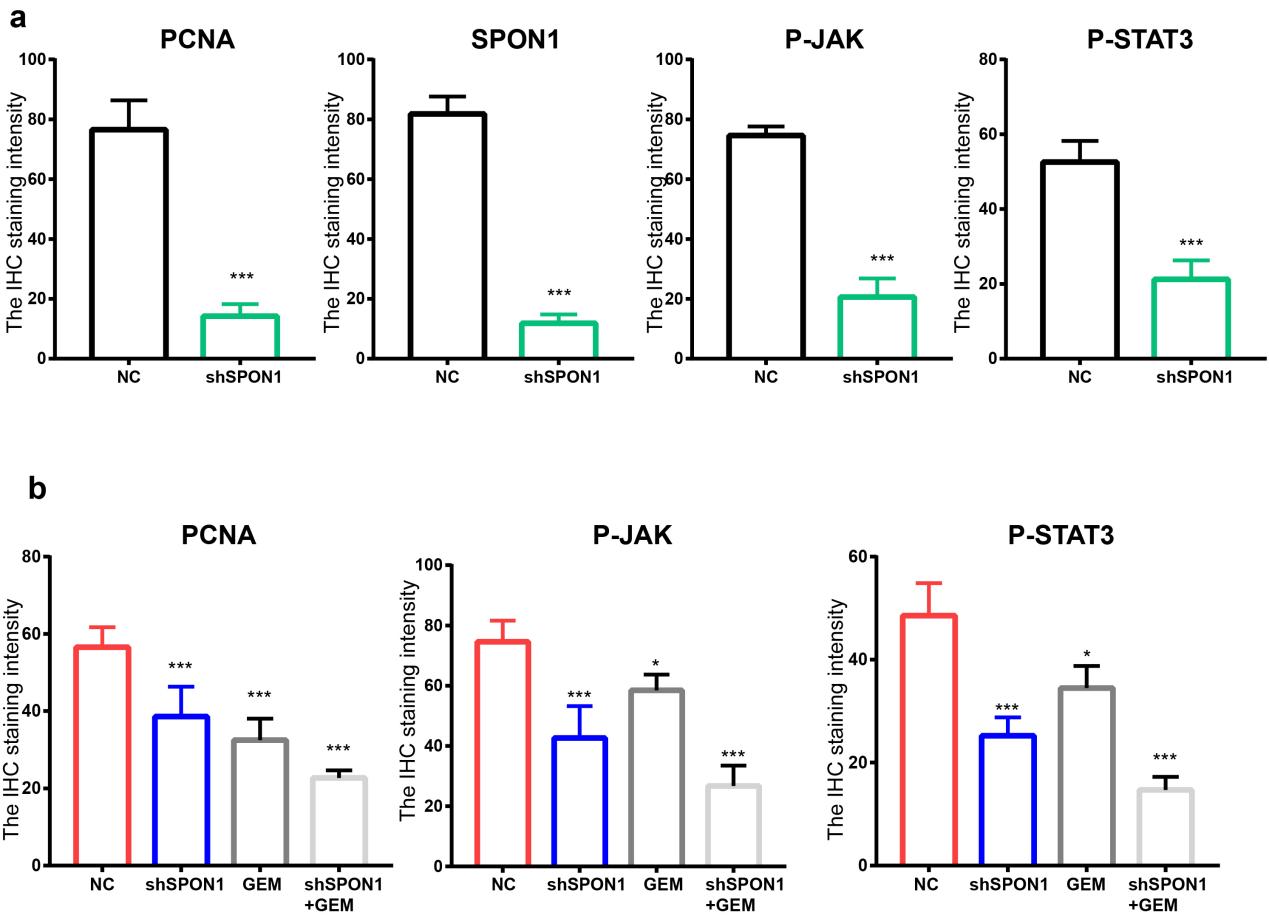


Supplementary figure 2 Targeting SPON1 inhibits JAK-STAT3 pathway in vivo

a-b.The statistical results of tumor section IHC staining intensity
